# Cultivar-Dependent Biosynthesis, Sustainable Recovery, and Industrial Applications of Tomato-Derived Carotenoids

**DOI:** 10.3390/plants15152371

**Published:** 2026-08-01

**Authors:** Han-Sol Kim, Byungwoon Goo, Seunghee Kim, Hee-Jin Kang, Jang-Seu Ki, Hah Young Yoo

**Affiliations:** 1 Department of Life Science, Sangmyung University, 20, Hongjimun 2-Gil, Jongno-Gu, Seoul 03016, Republic of Korea; biohansol0109@gmail.com (H.-S.K.);; 2 Department of Biotechnology, Sangmyung University, 20, Hongjimun 2-Gil, Jongno-Gu, Seoul 03016, Republic of Korea

**Keywords:** tomato, cultivar, carotenoids, biosynthesis, extraction, encapsulation

## Abstract

Tomato (*Solanum lycopersicum* L.) exhibits extensive cultivar diversity in fruit morphology, color, and bioactive compounds. These are closely associated with genetic background, ripening stage, and carotenoid metabolic regulation. Carotenoids, especially lycopene and β-carotene, are major determinants of tomato coloration and are also nutritionally and industrially important high-value-added antioxidants. Carotenoid profiles are regulated by changes in related biosynthetic pathways, including precursor supply, desaturation, isomerization, and cyclization. Key genes such as *PSY1*, *CRTISO*, *LCY-B* and *LCY-E* play central roles in carotenoid synthesis and accumulation, thereby determining color phenotypes. Mutations and genome-editing (CRISPR/Cas9) approaches can modify carotenoid metabolic flux and develop cultivars with improved nutritional traits. Furthermore, advanced and sustainable recovery strategies for carotenoids (ultrasound-, microwave-, enzyme-, high-pressure-, deep eutectic solvent-, and supercritical fluid-assisted extraction methods) have been developed for efficient pigment extraction. In this review, we compare the physiological and molecular characteristics of diverse cultivars and traits of advanced bioprocesses in terms of carotenoid extraction efficiency and solvent safety. Finally, we discuss various encapsulation techniques that improve product stability and storage performance in industrial applications. Taken together, we review practical strategies combining cultivar-based carotenoid production, sustainable recovery, and product stabilization.

## 1. Introduction

Tomato (*Solanum lycopersicum* L.) is one of the most important horticultural crops cultivated worldwide. It is consumed as both fresh fruit and in various processed products such as paste, ketchup, and juice. Global tomato production exceeded 188 million tons in 2024, increasing about 20% from 2010 [1]. It contributes to global nutritional security and the development of value-added food products. Tomato cultivars exhibit extensive diversity in fruit size, shape, color, texture, and functional compound composition [2]. Through intensive breeding, these cultivar-dependent traits have been improved in terms of nutritional quality [3,4], shelf life [5], yield [3], and stress resistance [3,6], thereby supporting the development of commercially valuable tomato varieties [7]. Among these traits, the color phenotypes of fruits and peels are strongly associated with differences in carotenoid composition. These visual characteristics are important for consumer preference and market segmentation and can also serve as phenotypic indicators of underlying metabolic and genetic variation [8,9]. In tomato, carotenoid biosynthesis and accumulation are regulated by the presence, absence, mutation, and transcriptional regulation of the pathway-related genes.

Carotenoids are lipophilic pigments found in plants, algae, and certain microorganisms, with more than 1100 known structures reported [10]. Tomato fruits mainly accumulate lycopene and β-carotene, although their profiles vary with cultivar and ripening stage [2,11]. The composition of these carotenoids is strictly regulated by genetic and environmental factors. Specifically, environmental conditions such as light, temperature, salinity, and cultivation practices significantly alter biosynthetic gene expression profiles. Cultivar-specific genotypes determine carotenoid profiles, which are further modified during fruit development and ripening [12,13]. These compounds contribute to fruit coloration and provide various biological functions, including provitamin A activity, antioxidant capacity, and supportive roles in visual health. Accordingly, carotenoids have attracted considerable attention as high-value-added materials with significant potential in the food and beverage, functional food, and pharmaceutical and cosmetic industries [14,15]. The global carotenoid market is expected to increase from USD 3262 million in 2024 to USD 5221 million by 2033, with a compound annual growth rate of 5.4% [16]. In addition, industrial demand for plant-derived carotenoids is increasing due to growing consumer preference for natural and clean-label ingredients. In this regard, natural carotenoid resources have shown a high growth rate of 5.7%, highlighting their potential as alternatives to synthetic carotenoids [16].

Annually, large amounts of tomato byproducts are produced during the processing of tomato products. Such byproducts are mainly composed of skins, seeds, and fibrous tissues, which are also called tomato pomace. Approximately 4–7% of fresh tomato weight remains as pomace, producing 8.5 million tons of byproducts [17,18]. Recently, it has been recognized as a promising raw material for sustainable carotenoid recovery, and diverse advanced extraction strategies improve recovery efficiency while reducing environmental impacts [19,20,21]. Accordingly, an understanding of carotenoid profiles and their underlying biosynthetic mechanisms across tomato cultivars is important for establishing strategies for carotenoid industrialization. It can provide a scientific basis for cultivar selection and development, including metabolic engineering to maximize carotenoid yield and quality [22]. In addition, various factors, including solvent safety, energy consumption, thermal stability, and process scalability, should be considered to ensure feasibility and industrial applicability [23,24]. Sustainable processing strategies have increasingly been highlighted to address regulatory requirements and environmental concerns associated with large-scale production.

Previous reviews on tomato carotenoids have mainly focused on individual topics such as carotenoid biosynthesis, lycopene accumulation, and health benefits [2,18,25]. However, studies comprehensively reviewing cultivar-specific carotenoid profiles, genetic determinants of fruit color, advanced extraction and stabilization strategies are still limited. This review provides novel strategies to increase industrial utilization by linking tomato byproducts with green extraction techniques and stabilization processes. Here, we review cultivar- and ripening-dependent differences in tomato carotenoid content and outline the biosynthetic pathways and major genetic determinants. In addition, diverse advanced extraction technologies are compared, with an emphasis on their performance and industrial scalability. Furthermore, encapsulation strategies and their industrial applications are discussed in relation to carotenoid stabilization and bioaccessibility. Lastly, we suggest an integrated strategy for expanding the applicability of tomato-derived carotenoids in the food, nutraceutical, pharmaceutical, and cosmetic industries.

## 2. Color and Carotenoid Profile of Tomato Cultivars

Tomato cultivars exhibit considerable diversity in morphology, including cherry, plum, beefsteak, and pear types, as well as in growth habits, such as determinate and indeterminate types, and production use, including fresh-market and processing cultivars. Fruit size, color, sugar content, texture, and functional compound composition vary greatly depending on the cultivars and ripening stages [11,26]. In particular, various cultivars with yellow, orange, pink, red, and purple fruit colors have been developed, and these color phenotypes reflect differences in carotenoid metabolism and pigment accumulation driven by the genetic variation [25,27,28,29]. In ripe red tomato fruits, lycopene is the predominant carotenoid, accounting for approximately 90% of total carotenoids, followed by β-carotene, which generally accounts for approximately 5–10% [30]. Colorless carotenoid precursors, such as phytoene and phytofluene, are also present, whereas lutein, α-carotene, γ-carotene, and ζ-carotene are detected at relatively low or trace levels [2,11].

Immature green tomato fruits contain chloroplast-rich cells that are responsible for photosynthesis. Lutein, a yellow xanthophyll pigment located in the thylakoid membranes of chloroplasts, contributes to the structural stability of light-harvesting complex II (LHCII) and protects photosynthetic tissues from excessive light stress [31]. During ripening, chloroplasts are converted into chromoplasts, which function as the main storage organelles for carotenoids [32]. Specifically, ethylene-mediated ripening promotes the degradation of thylakoid structures and LHCII proteins within chloroplasts [33], which is accompanied by the oxidative and metabolic degradation of lutein [34]. As a result, chloroplasts are transformed into chromoplasts capable of accumulating lycopene (bright red pigment) and/or β-carotene (reddish-orange pigment), whereas lutein content gradually decreases during ripening [32,35]. Taken together, the color of tomato fruits and peels is determined by variations in carotenoid composition and concentration, which are strongly dependent on cultivar characteristics and ripening stage (Table 1). In this table, direct quantitative comparisons between the cultivars are limited, since the data were obtained using different samples (cultivars, ripening stages, and cultivation environments) and carotenoid extraction and analysis methods. However, it is clear that the color phenotype of the fruit is significantly influenced by the predominant carotenoids or their composition. For example, high concentrations of lycopene and β-carotene are commonly observed in red, pink, orange, and purple tomatoes, whereas very low levels or almost no accumulation of these carotenoids is detected in immature green or yellow tomatoes. Among these color groups, purple cultivars tend to show relatively high average carotenoid contents; however, this is because certain cultivars (e.g., Cherokee Purple) tend to synthesize high levels of lycopene rather than anthocyanin. In addition, comparisons between mature red or orange tomatoes and immature green tomatoes have shown that lutein remains at higher levels in immature fruits [36].

The transition of tomato fruit color from green to yellow, orange, pink, red, or purple results from combined pigment changes in both the flesh and peel at the cellular level [8]. Common red tomato cultivars, such as San Marzano and Roma, accumulate high concentrations of lycopene in the chromoplasts of the fruit flesh, while naringenin chalcone, a flavonoid pigment, accumulates in the peel and gives it a yellowish appearance. Consequently, the bright red phenotype is produced through the visual layering of a yellow peel over red flesh. Pink tomato cultivars, such as Momotaro and Brandywine, also accumulate lycopene in the fruit flesh, but the peel remains transparent due to the absence or marked reduction in naringenin chalcone, resulting in a pink appearance [37]. Orange tomatoes can be divided into two representative types, β-carotene-rich types, such as Sun Sugar and Orange Wellington, and tangerine-type cultivars, such as Tangerine and Kellogg’s Breakfast [38]. In β-carotene-rich orange tomatoes, lycopene is efficiently converted into β-carotene, leading to an orange rather than red phenotype [39]. In contrast, tangerine-type tomatoes exhibit impaired conversion of prolycopene, a *cis*-lycopene isomer, into all-*trans*-lycopene, resulting in the accumulation of orange-colored carotenoid intermediates [40]. Although the major carotenoids responsible for the orange color differ between these two types, both ultimately exhibit orange fruit phenotypes.

**Table 1:** Carotenoid contents in various tomato cultivars, colors, and ripening stages.

Cultivar	Color	Carotenoid Content (mg/kg Dry Weight)			References
		Lycopene	β-Carotene	Lutein	
N1480E	Red	152.6 ± 3.5	51.9 ± 2.5	2.3 ± 0.3	[41]
TI00-0028BCAA	Red	141.4 ± 3.2	51.9 ± 4.0	2.7 ± 0.3	[41]
Cherry cereja ^b^	Red	69.2 ± 0.9	2.4 ± 0.0	1.6± 0.0	[42]
Rio Grande ^a^	Red	97.0 ± 9.8	105.0 ± 12.0 ^b^	N.D.	[43]
HLY 13 ^a^	Red	220.0 ± 8	231.0 ± 9.0 ^b^	N.D.	[43]
Lyco 2 ^a^	Red	184.0 ± 8	199.0 ± 7.0 ^b^	N.D.	[43]
Cv Donald	Red	95.0 ± 11.0	3.4 ± 0.2	0.3 ± 0.0	[44]
Cv Incas	Red	93.0 ± 5.0	2.3 ± 0.4	0.4 ± 0.0	[44]
Cv Kalvert	Red	202.0 ± 8.0	3.6 ± 0.2	0.7 ± 0.0	[44]
Cv Hly18	Red	195.0 ± 12.0	3.4 ± 0.2	1.8 ± 0.1	[44]
TI01-0044BABAA	Pink	132.9 ± 4.2	43.7 ± 3.0	2.0 ± 0.2	[41]
Market at Huachinango	Pink	140.8 ± 3.1	39.4 ± 1.9	2.1 ± 0.2	[41]
Rio Grande ^a^	Orange-red	26.7 ± 1.4	32.2 ± 1.6 ^b^	N.D.	[43]
HLY 13 ^a^	Orange-red	53.3 ± 0.9	61.9 ± 1.0 ^b^	N.D.	[43]
Lyco 2 ^a^	Orange-red	52.4 ± 0.6	60.5 ± 0.8 ^b^	N.D.	[43]
Cv Donald	Orange-red	50.0 ± 4.0	2.3 ± 0.1	0.3 ± 0.1	[44]
Cv Incas	Orange-red	51.0 ± 2.0	2.4 ± 0.2	1.2 ± 0.0	[44]
Cv Kalvert	Orange-red	58.0 ± 4.0	3.2 ± 0.4	1.7 ± 0.2	[44]
Cv Hly18	Orange-red	122.0 ± 9.0	3.9 ± 0.5	1.8 ± 0.1	[44]
V186A	Orange	51.7 ± 1.2	151.2 ± 5.1	0.7 ± 0.2	[41]
Jaune Flammee	Orange	52.2 ± 0.4	182.6 ± 4.1	1.6 ± 0.2	[41]
Cherry pera naranja ^b^	Orange	N.D.	0.5 ± 0.0	0.6 ± 0.0	[42]
Orange ^b^	Orange	30.5 ± 0.6	1.9 ± 0.2	0.4 ± 0.1	[42]
Cv Donald	Green	0.2 ± 0.01	0.6 ± 0.01	3.2 ± 0.3	[44]
Cv Incas	Green	0.2 ± 0.01	1.0 ± 0.02	5.6 ± 0.6	[44]
Cv Kalvert	Green	0.4 ± 0.01	8.3 ± 0.7	8.4 ± 0.6	[44]
Cv Hly18	Green	0.4 ± 0.01	10.4 ± 0.4	14.6 ± 3	[44]
Cherry amarillo	Yellow	N.D.	12.0 ± 0.0	10.0 ± 0.0	[42]
Green Zebra ^b^	Yellow	N.D.	11.0 ± 1.0	8.0 ± 1.0	[42]
Wapsipinicon peach	Yellow	48.4 ± 1.6	47.5 ± 1.5	3.9 ± 0.3	[41]
V118	Purple	184.4 ± 2.6	47.8 ± 2.6	2.7 ± 0.3	[41]
Cherokee Purple	Purple	153.2 ± 2.5	44.1 ± 2.4	2.2 ± 0.2	[41]
O258AAAA	Purple	190.0 ± 3.8	52.5 ± 2.2	4.2 ± 0.4	[41]

^a^ Fresh wet weight (mg/kg wet weight); ^b^ carotenoids (mg equivalents per kg of fresh wet weight); N.D. (not detected).

Yellow tomato cultivars, such as Yellow Pear and Golden Jubilee, generally contain low levels of lycopene and β-carotene, and their color is mainly associated with trace amounts of yellow pigments such as lutein and violaxanthin [28,41,42]. Purple tomatoes represent a distinct coloration pattern. For example, the cultivar Indigo Rose accumulates water-soluble anthocyanins in the vacuoles of peel cells, whereas Cherokee Purple exhibits a purple to brownish phenotype due to the combined effects of lycopene and residual chlorophyll pigments [41]. Tomato coloration is therefore not explained by the presence or absence of a single pigment. Instead, it depends on the combined effects of pigment accumulation in the flesh and peel, chlorophyll degradation, and changes in metabolic flux during fruit ripening [25,45]. These cultivar-dependent color traits provide useful phenotypic information for understanding carotenoid accumulation and the genetic regulation underlying a fruit quality.

## 3. Carotenoid Biosynthetic Pathway

**Figure 1:**
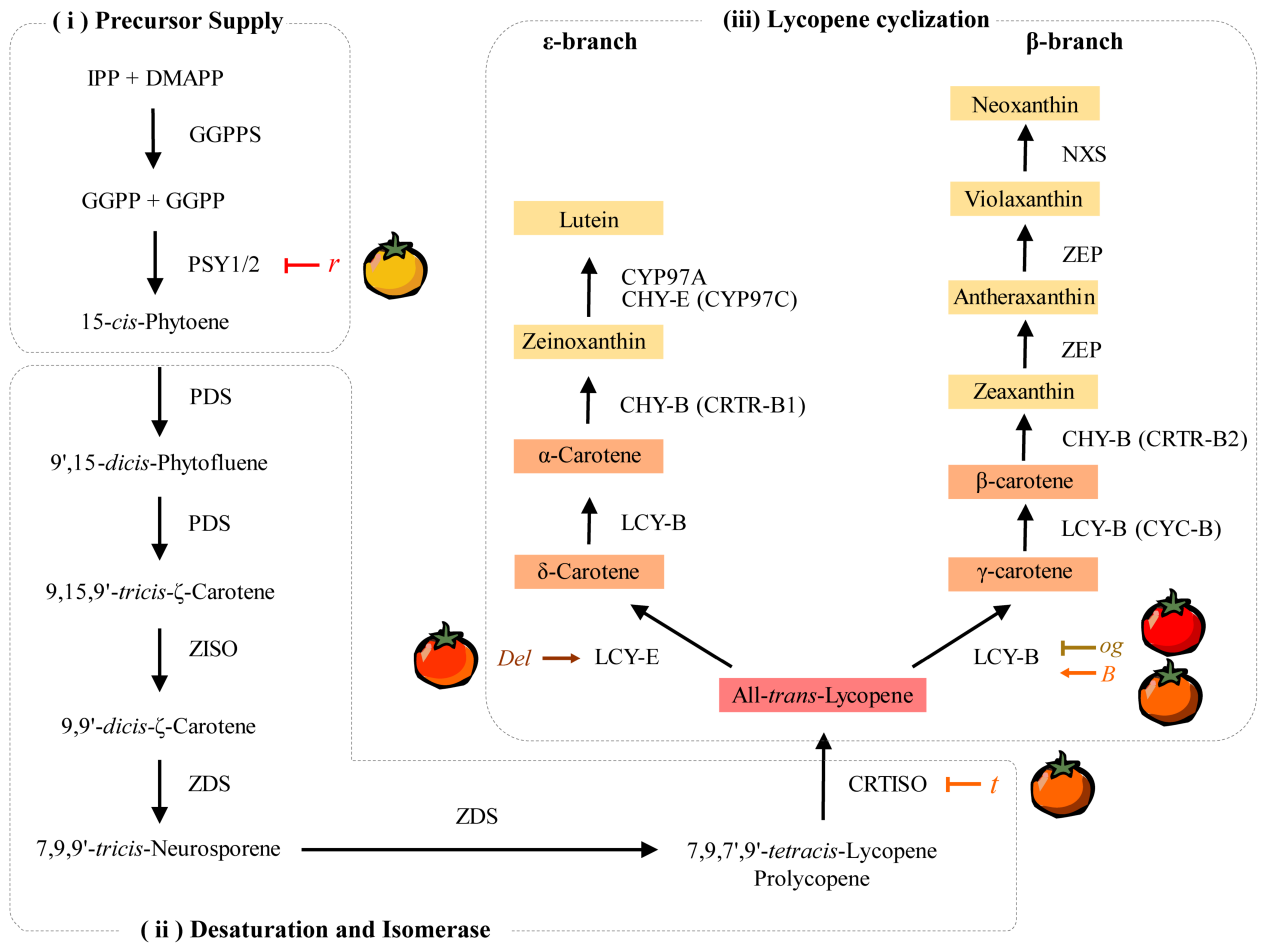
Schematic representation of carotenoid biosynthetic pathway. The enzymes and mutants are marked on the pathway in italics. Enzymes specifically found in tomato are written in parentheses. IPP: isopentenyl diphosphate, GGPP: geranylgeranyl diphosphate, GGPPS: GGPP synthase, PSY: phytoene synthase, PDS: phytoene desaturase, ZISO: ζ-carotene isomerase, ZDS: ζ-carotene desaturase, CRTISO: carotenoid isomerase, LCY-B: lycopene β-cyclase, CYC-B: chromoplast-specific lycopene β-cyclase; LCY-E: lycopene ε-cyclase, CHY-B: β-carotene hydroxylase, CHY-E: ε-carotene hydroxylase, CRTR-B1/2: β-carotene hydroxylase 1/2, ZEP: zeaxanthin epoxidase, NXS: neoxanthin synthase, CYP97A/C: cytochrome P450 enzymes. The malfunction and/or loss of *PSY1*, *CRTISO*, and *LCY-B* leads to Yellow Flesh (*r*), Tangerine (*t*), and Old-Gold (*og*) phenotypes. Overexpression of *LCY-B* and *CYC-B* results in Delta (*Del*) and Beta (*B*) phenotypes.

Tomato fruit undergoes dynamic color changes during ripening. These are tightly regulated by carotenoid metabolic flux and the coordinated expression of related genes. Carotenoid biosynthesis in tomato is broadly divided into three major stages: precursor supply, desaturation and isomerization, and lycopene cyclization (Figure 1). First, the carbon backbone is synthesized through the methylerythritol 4-phosphate (MEP) pathway. Isopentenyl diphosphate (IPP) and its isomer dimethylallyl diphosphate (DMAPP) are condensed by geranylgeranyl diphosphate synthase (GGPPS) to produce geranylgeranyl diphosphate (GGPP), the precursor for carotenoid biosynthesis [2,9,11]. Phytoene synthase (PSY) catalyzes the first step, which condenses two GGPP molecules to form 15-*cis*-phytoene [29]. Three *PSY* paralogs (*PSY1*, *PSY2*, and *PSY3*) show tissue-specific expression patterns [46]. *PSY1* is predominantly expressed during fruit ripening and plays a key role in lycopene accumulation, whereas PSY2 mainly functions in leaves and other green tissues, contributing to the synthesis of photosynthetic accessory pigments. *PSY3* is mainly expressed in roots under stress conditions and is rarely detected during fruit development and ripening [46].

In addition, carotenoid biosynthesis and accumulation are controlled by ripening-related transcriptional regulators [47]. There are three major ripening regulator genes, including tomato ripening inhibitor (*rin*) [48], non-ripening (*NOR*) [49], and colorless non-ripening (*Cnr*) [50]. These regulators promote fruit softening and the transition from immature green to red. Accordingly, mutations in these genes cause fruits to remain firm and retain white-to-green coloration [50]. Also, tomato AGAMOUS-LIKE 1 (TAGL1) promotes fruit development and maturation [51], while AP2a regulates the maturation rate through negative feedback [52]. Notably, SlMYB transcription factors turn on and off various pigmentation pathways, such as flavonoids, anthocyanin, and carotenoids, depending on the type. For example, naringenin chalcone synthesis is regulated by *SlMYB12* [37], and anthocyanin synthesis is activated by *SlMYB75* introduced by genetic engineering [53]. Such transcription factors modulate the major genes of carotenoid synthesis, affecting the metabolic flow and pigment composition in tomato.

In the next stage, phytoene undergoes sequential desaturation and isomerization reactions to form a conjugated double-bond structure, which is responsible for visible light absorption and pigment coloration [14,15]. Phytoene desaturase (PDS) catalyzes two desaturation reactions that convert phytoene into ζ-carotene through phytofluene intermediates [54]. The resulting intermediates are mainly present as *cis*-isomers and are subsequently converted by ζ-carotene isomerase (ZISO). Thereafter, ζ-carotene desaturase (ZDS) catalyzes additional desaturation reactions, leading to the formation of neurosporene and ultimately lycopene [55]. Carotenoid isomerase (CRTISO) then converts tetra-*cis*-lycopene, also known as prolycopene, into all-*trans*-lycopene, which is the major red carotenoid accumulated in ripe tomato fruits [56].

The all-*trans*-lycopene is further metabolized through two major cyclization branches (ε-branch and β-branch) depending on the lycopene cyclase. In the ε-branch, lycopene ε-cyclase (LCY-E) introduces an ε-ring at one end of all-*trans*-lycopene to form δ-carotene. Subsequently, lycopene β-cyclase (LCY-B) introduces a β-ring to δ-carotene to produce α -carotene [57]. On the other hand, the β-branch leads to the synthesis of β-carotene and xanthophyll derivatives, some of which serve as precursors for plant hormone abscisic acid (ABA). LCY-B and/or chromoplast-specific β-cyclase (CYC-B) converts all-*trans*-lycopene into γ-carotene and then β-carotene through the sequential addition of β-rings [58,59]. β-carotene is hydroxylated by β-carotene hydroxylase (CHY-B) to form β-cryptoxanthin and zeaxanthin. Zeaxanthin is further converted into antheraxanthin and violaxanthin by zeaxanthin epoxidase (ZEP), and violaxanthin is converted into neoxanthin by neoxanthin synthase (NXS) [60]. These xanthophylls serve as precursors for ABA biosynthesis through oxidative cleavage reactions by 9-*cis*-epoxycarotenoid dioxygenase (SlNCED1).

Plant hormone ABA serves as a developmental clock that completes cell growth and determines the initiation of the ripening stage. When the fruit changes from immature green to mature green status, the ABA concentration inside tomato increases rapidly, while auxin and gibberellin decrease [61]. Accumulated ABA signals activate the transcription factors SlAREB1, promoting the expression of *PSY1*, which initiates lycopene synthesis [62]. At the same time, ABA and ethylene signaling are associated with *SlACS2* and *SlACS4* expression, resulting in an explosive production of system II ethylene [63]. The promoted ethylene signal activates the network of ripening regulators, *Rin* and *NOR* genes [64]. In addition, the ABA signal induces the expression of the STAY-GREEN 1 (*SlSGR1*) gene, which degrades chlorophyll pigments in chloroplast [65]. As a result of these multifaceted molecular controls, photosynthetic chloroplasts are converted to chromoplasts, which accumulate large amounts of carotenoids. To sum up, hormones such as ABA and ethylene induce fruit maturation by regulating carotenoid metabolic pathways.

The development and maturation of tomato are also affected by several environmental and cultivation conditions. In particular, environmental factors such as light irradiance, temperature, salinity, and moisture [66,67], available nutrients [68], and horticultural practices [22] are key variables that alter the balance of carotenoid composition. Among those, high-temperature stress in particular is a major cause of inhibiting lycopene accumulation, resulting in a decrease in color and nutritional quality. For example, temperatures above 40 °C significantly decrease the mRNA expression levels of *SlPSY1* and *SlGGPS2*, thereby blocking the carotenoid metabolic flow [69]. As a result, the carotenoid profile of stressed tomato shifted from lycopene to β-carotene, reducing the commodity value [69]. Due to recent intensifying climate change causing recurrent high-temperature stress and water shortages [70], building a sustainable tomato production system is an essential challenge for future agriculture. In this regard, a smart farm system can precisely control environmental factors, and, in fact, artificial intelligence (AI)-integrated smart farms can increase crop yield by 10% and net profit by 92% [71]. In addition, for stable mass production under open-field conditions, cultivar development through molecular breeding is also required, thereby securing high-quality carotenoid resources [72].

Overall, carotenoid composition among tomato cultivars is determined by precursor supply and desaturation, isomerization, and cyclization reactions [2,11]. Sequential enzymatic reactions control the metabolic flux, which are significantly affected by the overexpression, suppression, or mutation of key genes [28,29]. Their transcription levels are also regulated by hormones [62,65] and diverse environmental factors [22,66]. For this reason, genetic engineering is a key strategy for cultivar development that can enhance the carotenoid profile and yield [72]. Therefore, tomato carotenoid biosynthesis should be understood as an integrated system controlled by a broad regulatory framework. Such information provides a useful basis for selecting or engineering cultivars with desirable carotenoid profiles for industrial applications.

## 4. Engineering Carotenoid Biosynthetic Gene for Cultivar Improvement

Carotenoids in tomato flesh and peel are key phenotypic indicators of fruit coloration. Their composition is regulated by metabolic flux and related enzyme activities and their encoding genes. Color phenotypes associated with gene mutations include wild-type red tomato and mutants such as Yellow Flesh (*r*), Tangerine (*t*), Delta (*Del*), Beta (*B*), Old-Gold (*og*), and green flesh (*gf*). The characteristic red color of the mature wild-type mainly results from an extensive accumulation of all-*trans*-lycopene, which accounts for 80–90% of the total carotenoid pool [30]. Lycopene accumulation requires continuous precursor supply and coordinated desaturation, isomerization, and cyclization. However, when genetic variations disrupt or enhance these key checkpoints, carotenoid flux changes and distinct flesh or peel colors appear.

Gene mutations causing enzymatic dysfunction include nucleotide sequence change, large sequence insertion or deletion, and start/stop codon mutation. For example, mutations in *PSY1* impair its function and reduce phytoene formation [29,73]. Since PSY1 catalyzes the first step of carotenoid biosynthesis, this dysfunction results in a marked decrease in lycopene accumulation. Consequently, *PSY1* mutations lead to the Yellow Flesh (*r*) phenotype, which is characterized by an absence or severe reduction in red pigmentation [28]. For example, *r3756*, a *PSY1* mutant generated by ethyl methanesulfonate (EMS) mutagenesis in the standard tomato cultivar ‘M82’, has been widely used to study carotenoid metabolism. Meanwhile, the transparent peel or pink fruit phenotype is associated with the loss of SlMYB12, a transcription factor that regulates flavonoid biosynthesis in the peel [74]. SlMYB12 loss blocks the accumulation of naringenin chalcone, resulting in a transparent peel that allows the red flesh to appear pink.

The *t* phenotype is a recessive mutant characterized by an orange color, which is caused by *CRTISO* gene mutation [55]. The loss of CRTISO activity inhibits the conversion of tetra-*cis*-lycopene into all-*trans*-lycopene, leading to prolycopene accumulation and orange fruit. Meanwhile, distinct color phenotypes can also be induced by functional enhancement or gene overexpression [25]. For example, the *Del* phenotype shows increased expression of LCY-E, which is generally down-regulated during fruit ripening [75]. Sustained ε-cyclase activity redirects metabolic flux toward the ε-cyclization branch, promoting δ-carotene accumulation and a reddish-orange phenotype.

The *og* and *B* phenotypes show opposite regulatory effects on *CYC-B*, which encodes a chromoplast-specific β-cyclase [76,77]. In the *og* mutant, the loss of *CYC-B* function suppresses the conversion of lycopene into β-carotene, resulting in increased lycopene accumulation and a deep red fruit color. In contrast, a promoter mutation in the *B* mutant induces *CYC-B* overexpression, thereby accelerating the conversion of lycopene into β-carotene and producing an orange fruit phenotype [76]. In some cultivars, fruit coloration is also influenced by chlorophyll retention. The green flesh (*gf*) phenotype is caused by mutations in *SGR1*, which prevent chlorophyll degradation during ripening [78,79]. The persistence of chlorophyll, combined with lycopene accumulation, results in a brown to dark-colored phenotype [80].

Beyond natural and induced mutations, transgenic and genome-editing approaches have been employed to enhance carotenoid accumulation and introduce novel traits. A representative example is purple tomato developed by Norfolk Plant Sciences (Norwich, Norfolk, UK), in which the snapdragon (*Antirrhinum majus*) transcription factor genes Rosea1 (*AmRos1*) and Delila (*AmDel*) were introduced to activate anthocyanin biosynthesis [81,82]. Although this approach primarily targets anthocyanin accumulation rather than carotenoid biosynthesis, it demonstrates the potential of metabolic engineering to generate new pigment profiles and functional traits in tomato. Genetic engineering has also been applied to improve high-value compound production and agronomic traits, including shelf life, pest resistance, and environmental tolerance. For example, the Flavr Savr cultivar is a classic example of antisense technology, in which the suppression of polygalacturonase gene expression delays fruit softening during ripening [83].

Recently, genome-editing research has extended to reprogramming carotenoid metabolic fluxes using molecular technologies such as clustered regularly interspaced short palindromic repeats (CRISPR/Cas9), base and prime editing, and transcription activator-like effector nuclease (TALEN) [84]. These can shorten cultivar development and allow the precise modification of fruit quality, productivity, and environmental adaptability [72]. For example, the ‘Sicilian Rouge High GABA’ tomato is a representative CRISPR/Cas9-edited cultivar. Gene editing of SlGAD3 resulted in enhanced glutamate decarboxylase activity and increased γ-aminobutyric acid (GABA) accumulation [84]. Also, editing of the major carotenoid biosynthetic genes of *PSY1* and *CRTR-B2* modified the fruit and flower color phenotype [85]. Meanwhile, the elimination of *CYC-B* promoted early lycopene accumulation and improved cold resistance after harvesting [86]. Multiple CRISPR/Cas9 editing (*SGR1*, *LCY-E*, *LCY-B1*, and *LCY-B2*) using *Agrobacterium tumefaciens*-mediated transformation increased lycopene accumulation by up to 5.1-fold by promoting lycopene biosynthesis [87]. Similarly, a series of color phenotypes were derived according to multiplex editing combinations of *PSY1*, *MYB12*, and *SGR1* [88]. These examples indicate that gene editing can regulate carotenoid flux and facilitate the rapid development of tomato cultivars with improved nutritional and industrial benefits [88]. Nevertheless, several challenges remain, including off-target effects, GMO regulatory approval, and consumer acceptance. Given the increasing demand for nutrient-enriched crops and natural ingredients, gene editing is likely to remain an important tool in tomato breeding. For carotenoid-oriented breeding, visual phenotype assessment, quantitative carotenoid profiling, genotyping, and metabolomics should be combined to identify suitable varieties [28,29].

## 5. Carotenoid Extraction from Tomato

Tomato-derived carotenoids can be extracted not only from edible fruit tissues but also from byproduct pomace [19,20,21]. In particular, large quantities of lycopene and β-carotene are also present in pomace, suggesting that byproduct-based recovery processes can provide both economic and environmental benefits [20]. However, carotenoids are highly sensitive to heat, light, oxygen, and oxidative stress because of their polyene structures containing multiple conjugated double bonds. Therefore, advanced extraction processes are required to improve carotenoid recovery while minimizing pigment degradation. Process optimization is also essential to maximize carotenoid yield and maintain chemical stability during extraction, concentration, and storage [89,90]. When designing carotenoid extraction processes from tomato and its byproducts, extraction yield, degradation control, solvent safety, energy consumption, process scalability, and product quality should be considered (Table 2).

### 5.1. Traditional and Advanced Carotenoid Extraction Methods

Maceration, Soxhlet extraction, and cold pressing have served as traditional methods for recovering lipophilic pigments from plant materials [15]. Maceration is a simple and inexpensive solid–liquid extraction method that can be performed at room temperature. This method has advantages such as operational simplicity and low initial investment. However, because mass transfer mainly depends on passive diffusion, carotenoid extraction efficiency is relatively low, requiring a long extraction time and high solvent consumption [19,91]. In addition, the prolonged exposure of carotenoids to light, oxygen, and processing solvents can also accelerate oxidative degradation and pigment bleaching [15,89,92].

**Table 2:** Comparative overview of conventional and advanced extraction methods for tomato-derived carotenoids. B/S: biomass–substrate ratio, CE: conventional extraction, DES: deep eutectic solvent, EAE: enzyme-assisted extraction, EL: ethyl lactate, HPAE: high-pressure-assisted extraction, MAE: microwave-assisted extraction, NADES: natural DES, PEF: pulsed electric field, RSM: response surface methodology, SFE: supercritical fluid extraction, UAE: ultrasound-assisted extraction.

Extraction Method	Representative Extraction Performance with References	Advantages	Limitations	Solvent Safety	Scalability	References
Maceration	Resource: Tomato processing waste peel Condition: RSM optimization: solvent/meal ratio 30:1 (*v*/*w*), particle size 0.15 mm, 50 °C, 8 min Yield: Lycopene: 19.8 mg/kg	Simple operation, low equipment cost, broad applicability for laboratory screening	High solvent consumption, long extraction time, pigment oxidation, low sustainability	Hexane, acetone, and ethyl acetate raise safety and environmental concerns	Technically scalable, but requires solvent recovery and safety management	[92]
	Resource: Dried tomato pomace powder Condition: Ethyl acetate, 25 °C, 120 min, solid/liquid ratio 1:46 g/mL Yield: Total carotenoids 505–505.3 mg/kg DW, β-carotene 123.8 mg/kg DW, lycopene 386.2 mg/kg DW					[20]
UAE	Resource: Tomato processing wastes (peel and seed) Condition: Hexane:methanol:acetone 2:1:1 + 0.05% BHT; solid/liquid 1:35 (*w*/*v*), 15 ± 5 °C, UAE at 50, 65, or 90 W for 1–30 min Yield: Lycopene 52.21–57.19 mg/kg DW, β-carotene 4.42–4.90 mg/kg DW	Enhances cell disruption and solvent penetration, shortens extraction time, operable under mild temperature conditions	Excessive ultrasound intensity may generate heat, oxidation, or degradation; performance is highly matrix- and condition-dependent	Compatible with ethanol, edible oils, SDS, and other greener solvents	Promising but sophisticated process optimization is required	[93]
	Resource: Industrial tomato byproduct Condition: Ethanolic pulsed UAE, particle size < 56 µm, solid:liquid ratio 2:25, duty cycle 30:30, 45 °C Yield: Lycopene 2586 mg/kg FDW, β-carotene 161 mg/kg FDW					[91]
	Resource: Tomato processing industry waste Condition: Ultrasound bath extraction Yield: Lycopene 420.8 µg/g DW Note: Conventional stirring extraction showed higher yield					[19]
MAE	Resource: Dried tomato pomace powder Condition: MAE: 150 W, S/L 1:30 g/mL, 50 °C, 30 min Yield: Total carotenoids 592.5 mg/kg DW Note: Time reduced from 120 min (CE) to 30 min (MAE)	Rapid heating, short processing time, improved cell disruption, reduced extraction time	Overheating induces carotenoid degradation, isomerization, requires process validation	High when combined with food-grade solvents	Uniform microwave energy distribution and thermal control are required for scale-up	[20]
Combined UAE–MAE	Resource: Dried tomato peels Condition: EL, 500 W microwave power, 600 W ultrasound power, 45 °C for 8 min Yield: Lycopene: 370.9 ± 20.9 mg/kg Note: 91% relative to Soxhlet (4 h)	Synergistic cell destruction, high yield, short time, compatibility with eco-friendly solvents	Excessive microwave power may cause thermal degradation or isomerization, process validation is required	EL or other food-compatible solvents	Integrated control of microwave and ultrasonic energy distributions is required for scale-up	[94]
PEF	Resource: Tomato peel and pulp Condition: PEF at 5 kV/cm for 90 µs, hexane:ethanol 50:50, 300 min Yield: Total carotenoids: 58.81 ± 9.44 µg/g FW Recovery: 39% increase compared to untreated control; reduced hexane usage from 45% to 30% without reducing yield	Non-thermal cell permeabilization, enhanced mass transfer, shorter time, potential reduction in solvent use	Efficiency depends on matrix (tomato pulp < peel), field strength and energy input optimization required	Can use green solvents (ethanol and EL), some studies still use hexane mixed solvents	High potential with a continuous pretreatment technology	[95]
	Resource: Industrial tomato byproduct peel Condition: PEF at 5 kV/cm, 5 kJ/kg, acetone or EL Note: Lycopene yield increased by 12–18%, extraction rate increased by 27–37%					[96]
HPAE	Resource: Dried tomato pomace powder Condition: HPAE, 650 MPa, ambient temperature, S/L 1:10 g/mL Pretreatment: stirring for 24 h Yield: Total carotenoids 84 mg/kg DW (HPAE), 277 mg/kg DW (HPAE + pretreatment) Recovery: 55% of carotenoids extracted by CE	Mild or non-thermal processing, reduced thermal degradation, cell disruption	HPAE single treatment is insufficient for high recovery, high equipment cost, suitable for pretreatment	High when combined with food-grade solvents or edible oils	Industrially scalable, but capital-intensive	[20]
EAE	Resource: Tomato processing waste peel Condition: Food-grade pectinolytic and cellulolytic enzyme, RSM optimization (30 °C, 3.18 h, enzyme load: 0.16 kg/kg) Recovery: Lycopene recovery increased 8- to 18-fold, respectively	Mild processing, selective cell wall degradation, reduced pigment degradation, food-grade compatibility	Enzyme cost, long pretreatment time, pH and temperature dependence, additional solvent or surfactant extraction steps required	High when food-grade enzymes and solvents (edible oils and EL) are used	More suitable for pretreatment or part of complex processing	[97]
	Resource: Tomato peels Condition: Viscozyme L pretreatment 1.4%, 52 °C, 92 min; extraction 25 °C, 30 min, rice bran oil, solid:oil 1:20 (*w*/*v*) Yield: Lycopene 3996 mg/kg DW					[98]
	Resource: Tomato waste Condition: EAE: pectinase/cellulase and EL; HPAE: 700 MPa, 10 min Yield: Total carotenoids 127 mg/kg DW; lycopene 89.4 mg/kg DW Recovery: EAE treatment increased 6-fold (carotenoids) and 10-fold (lycopene) compared to non-treated samples					[99]
DES/NADES	Resource: Tomato pomace Condition: Hydrophobic DES (menthol:butyric acid 1:2), 65 °C, 50 min, 1/10 as B/S ratio Yield: Total carotenoids 300 µg/g DW, β-carotene 24 µg/g DW, lycopene 52 µg/g DW	Switchable solvent, Low volatility, high affinity for carotenoids, reduced dependence on toxic solvents	High viscosity, high separation difficulty, and regulatory acceptance remain challenges	NADES provides better food compatibility	Solvent recovery and separation process validation is required	[21]
	Resource: Tomato waste powder Condition: Choline chloride:1,3-butanediol (1:5), S/L 1:20 (*w*/*v*), 12 min, 65 °C, radiation frequency 37 Hz, ultrasound power 70% Yield: Lycopene 215.1 ± 4.3 µg/g DW, β-carotene 206.9 ± 3.3 µg/g DW, tocopherol 130.9 ± 9.0 µg/g DW					[100]
SFE	Resource: Tomato processing byproduct Condition: Supercritical CO_2_ 500 mL, 86 °C, 34.47 MPa, flow rate 2.5 mL/min Yield: Lycopene 7.19 µg/g Recovery: Lycopene recovery 61.0%	Non-toxic CO_2_, low oxygen exposure, solvent-free extracts, high selectivity, suitable for premium products	High equipment cost, high-pressure operation, optimization of pressure, temperature, and co-solvents is required	CO_2_ and food-grade solvents	Industrially scalable, but capital-intensive, nutraceutical, pharmaceutical, and high-value food applications	[101]
	Resource: Tomato processing byproduct peel Condition: Supercritical CO_2_; 300 bar, 80 °C, flow rate 0.792 kg/h, particle size 0.345 mm, 130 g CO_2_/g matrix Recovery: Recovered 80% of lycopene and 88% of β-carotene					[102]

The Soxhlet extraction method repeatedly exposes samples to fresh solvent through solvent circulation, thereby improving carotenoid recovery efficiency. However, the high-temperature conditions used in this process can promote the thermal degradation of pigments, particularly lycopene and β-carotene [103]. Thermal stress can also isomerize all-*trans* carotenoids, which are the most stable forms in nature, into *cis*-isomers, resulting in reduced pigment quality and antioxidant capacity [91,92,93]. In addition, the excessive use of toxic organic solvents and low energy efficiency are not suitable for green and sustainable production systems [21]. Therefore, there is growing interest in innovative extraction technologies to reduce solvent use, processing time, and energy input [20,90].

**Figure 2:**
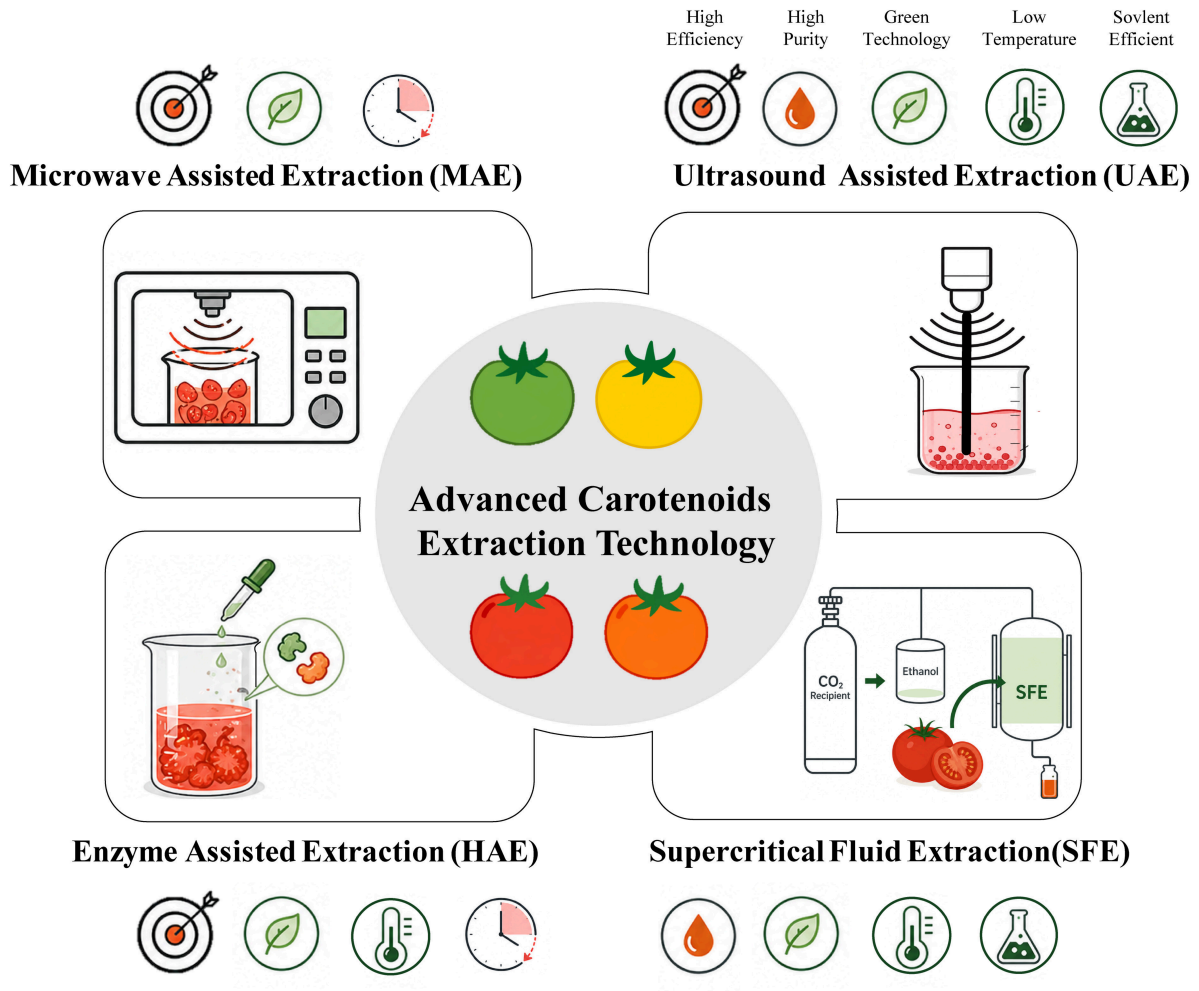
Schematic overview of major advanced extraction techniques used for the recovery of carotenoids from tomato and its byproducts. The icons of each method summarize key advantages such as high extraction efficiency, purity, reduced processing time, environmentally friendly technology, low-temperature conditions, and improved solvent efficiency.

To overcome the limitations of conventional extraction methods, various advanced strategies have been developed for carotenoid recovery from tomato and tomato-derived byproducts (Figure 2). Efficient extraction from tomato byproducts generally requires the disruption of rigid cell wall structures and improvement of solvent penetration using food-grade or eco-friendly solvents [104]. Cell wall disruption techniques can be broadly classified into physical, electromagnetic, electrical, and biochemical approaches. Physical and electromagnetic-assisted methods include high-pressure-assisted extraction (HPAE), ultrasound-assisted extraction (UAE), and microwave-assisted extraction (MAE) [105]. HPAE can efficiently disrupt tissues and adapt to continuous-flow systems for industrial-scale processing; however, there are potential challenges with heat generation and oxidative degradation. UAE uses acoustic cavitation to mechanically disrupt cell walls, enhance solvent penetration, and extract pigments under mild temperature conditions. Recent studies have optimized pulsed ultrasound conditions to balance energy use and carotenoid yield and stability [91,93,106].

MAE heats intracellular moisture by dielectric heating, thereby accelerating cell disruption and shortening extraction time. However, excessive microwave energy or prolonged exposure can cause thermal degradation and isomerization [19,89,101]. For example, MAE treatment at 150 W and 50 °C for 20 min achieved a carotenoid yield of 592.5 mg/kg dry weight, compared with 505.3 mg/kg dry weight using a conventional process [20]. Enzyme-assisted extraction (EAE) provides a milder biochemical pretreatment. Enzymes such as pectinase, cellulase, and hemicellulase degrade tomato cell wall polysaccharides and release carotenoids [107,108]. The application of food-grade pectinolytic and cellulolytic enzymes increased lycopene recovery by up to 18-fold, indicating the potential of EAE as a selective and safe strategy for carotenoid extraction [97].

### 5.2. Sustainable Carotenoid Recovery from Tomato Byproducts

Sustainable extraction has focused on replacing hexane, acetone, and other toxic organic solvents with eco-friendly alternatives. Deep eutectic solvents (DESs), natural deep eutectic solvents (NADESs), edible oil-based systems, and supercritical fluid extraction (SFE) are representative strategies for sustainable carotenoid recovery. Hydrophobic DES systems (e.g., menthol:butyric acid) as well as hydrophilic NADES formulations (e.g., choline chloride:lactic acid) have been investigated for carotenoid recovery from byproducts [21,109]. For example, optimized menthol–butyric acid solvent systems reached carotenoid yields of approximately 300 µg/g dry weight, demonstrating efficient carotenoid recovery [21]. Recently, NADESs have emerged as promising alternatives to DESs due to their low toxicity, biodegradability, and compatibility with food and nutraceutical applications depending on their solvent selection [100]. Specifically, surfactants and edible oils can also increase carotenoid recovery. Biodegradable anionic surfactants such as sodium dodecyl sulfate (SDS) [106] and edible oils such as sunflower and corn oils [104] are used in green extraction systems since they can solubilize lipophilic carotenoids and protect them from oxidative degradation. Bio-based eco-solvents, particularly terpenes such as *d*-limonene, ethyl lactate, and fatty acid methyl esters, exhibit excellent lipophilic affinity for non-polar carotenoids. While these strategies reduce the step for solvent evaporation, the solvents used in DESs/NADESs have a low vapor pressure. Due to the difficulty of removing solvents to isolate pure carotenoids, DESs containing extracts should consequently be added to the final products, or an additional post-processing recovery step is required.

SFE uses fluids above their critical temperature and pressure to separate target compounds selectively and efficiently [101,102]. Supercritical CO_2_ is widely used for carotenoid extraction because it is non-toxic, chemically inert, and easily removed after extraction. In tomato systems, optimal lycopene recovery (61.0%, equivalent to 7.2 µg/g) was achieved under conditions of 34.5 MPa and 86 °C [101]. The extraction efficiency of supercritical CO_2_ can be further improved by adjusting pressure, temperature, extraction time, and the use of co-solvents such as ethanol. Furthermore, recent studies have expanded the utility of tomato-derived extracts by incorporating them into new functional materials. Advanced extraction technologies aim to reduce reliance on toxic organic solvents while improving carotenoid recovery, stability, and process compatibility. This trend reflects a strategic shift toward sustainable, food-compatible, and industrially scalable recovery systems for tomato-derived carotenoids.

### 5.3. Encapsulation for Carotenoid Stabilization and Long-Term Storage

**Figure 3:**
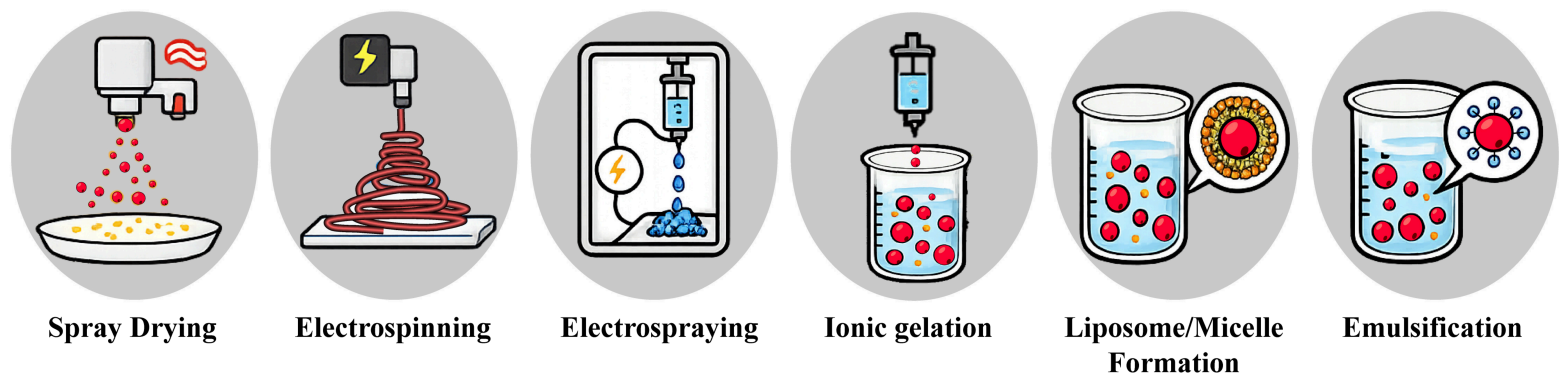
Schematic diagram of encapsulation techniques for carotenoids. The illustration highlights various methods used for carotenoid encapsulation, including spray drying, electrospinning, electrospraying, ionic gelation, liposome/micelle formation, and emulsification.

Encapsulation is used to protect carotenoids before they are added to final products or packaging systems [110,111]. Various encapsulation methods, including spray drying, freeze drying, electrospinning, electrospraying, ionic gelation, liposome and micelle formation, and emulsification, have been developed. These can enhance the stability, dispersibility, and bioavailability of extracted carotenoids (Figure 3). In general, microencapsulation (1–1000 µm) and nanoencapsulation (10–1000 nm) protect bioactive compounds from light, oxygen, heat, and chemical degradation. These systems can also mask undesirable taste and odor, improve handling properties, and convert carotenoid-rich extracts into powder or colloidal formulations. Among encapsulation strategies, microencapsulation and beadlet systems are generally used industrially since they can produce products in stable forms. On the other hand, despite the high encapsulation efficiency and improved dispersibility of nanoencapsulation and electrospinning, important limitations remain since these processes are complex and require strict safety evaluation and scale-up assessment [110,112,113,114].

Microencapsulation coats bioactive compounds in polymer matrices [111], and, specifically, beadlet technology has been widely applied in the carotenoid industry. In this technique, carotenoids are encapsulated within gelatin, starch, and other biopolymer matrices to form small spherical particles [112]. The starch-catch beadlet technique has shown high digestive stability, with recovery rates of 70–90% for carotenoids during digestion, except lycopene (50%) [115]. To date, microencapsulation has been the most widely used approach in industrial production, particularly when combined with spray drying to convert liquid carotenoids into stable powder forms [113]. Nanoencapsulation uses delivery systems such as liposomes, nanoemulsions, polymeric nanoparticles, and cyclodextrin-based complexes to control particle size at the nanometer scale [116]. Surface area and aqueous dispersibility are increased through nanoencapsulation, enhancing their gastrointestinal stability, absorption, and bioavailability. For example, El Basett et al. [116] encapsulated lycopene extracted from tomato peels into nanoemulsions using β-cyclodextrin and dextran, followed by spray drying. They achieved a high encapsulation efficiency of 96.2% and improved the physicochemical stability of lycopene, even under harsh temperature conditions [116]. Horuz et al. [117] also reported on encapsulation in zein nanofibers through electrospinning, improving lycopene retention and increasing antioxidant activity 11-fold. Also, conjugating extracted lycopene with TiO_2_ nanoparticles improved their photostability, reaching a loading efficiency of 95.0% [90].

Despite these advantages, various encapsulation technologies have limitations in industrial applications. For instance, spray drying and beadlet technologies are highly scalable and cost-effective, but they may have low bioaccessibility or weak protection compared to nanoscale systems [112,113,115]. Although nanoemulsion and nanofiber electrospinning can improve encapsulation efficiency, dispersibility, lycopene preservation, and antioxidant performance, they have problems such as process complexity, high equipment cost, particle size control, and evaluation for regulatory safety and scale-up [110,114,116,117]. Therefore, not only efficiency, but also storage stability, biological accessibility, food-matrix compatibility, production cost, and industrial scalability must be compared to select encapsulation platforms for carotenoid commercialization.

## 6. Industrial Applications and Future Perspectives of Carotenoids

Carotenoids are widely utilized in various industrial sectors, including feed, food, functional food, pharmaceutical, and cosmetic industries (Table 3). Tomato lycopene extracts and concentrates have been commercialized as oleoresin, powder, and water-dispersible formulations [14]. In the feed industry, carotenoids are mainly used as dietary additives to enhance coloration and physiological performance in fish and crustaceans [118]. Similarly, for food products, β-carotene, lycopene, and lutein have been used as natural colorants, and their commercial importance is rapidly increasing. This trend is mainly driven by growing consumer preference for high-value-added, clean label, and natural ingredients [15,104]. In functional food and nutraceutical applications, lycopene is attracting attention for its potential roles in antioxidant activity and cardiovascular health improvement. β-Carotene is also an important provitamin A precursor [119], whereas lutein and zeaxanthin are essential for maintaining macular health and visual function and are therefore key ingredients for eye health products [120]. Beyond nutritional applications, carotenoids have also been extensively investigated in the pharmaceutical and cosmetic sectors as bioactive compounds associated with oxidative stress regulation, photoprotection, anti-aging effects, and anti-inflammatory activity [119,121]. In particular, lycopene has been applied as an antioxidant ingredient for protecting skin from ultraviolet-induced oxidative damage, whereas lutein has been studied for its protective effects against blue-light-induced stress [122]. These biological properties support the use of carotenoids as functional ingredients in dietary supplements, topical formulations, and cosmeceutical products.

Given the wide range of industrial applications of carotenoids, it is essential to maintain the quality and stability of products during processing, storage, and distribution. In this regard, emulsification, encapsulation, and protective packaging are essential to improve their stability, dispersibility, and bioavailability [14,15]. Specifically, the commercialization of functional foods is subject to strict regulatory requirements concerning the safety of new technologies, quality control, and consumer protection. From a regulatory perspective, tomato-derived carotenoid products should be evaluated for their biological activity as well as solvent and processing aid safety, residual solvent control, oxidation stability, and encapsulating carrier safety [14,15,110,114]. Therefore, food-grade solvents, supercritical CO_2_, biodegradable surfactants, and biopolymer-based encapsulation systems have the advantage of being better compatible with food, nutritional supplements, and cosmetic applications than conventional processes [15,104,106,123].

**Table 3:** Potential industrial applications of carotenoids.

Industry	Functions or Applications	Ref.
Feed/ Aquaculture	Tomato total carotenoid extract: Strengthening pigmentation and antioxidant in aquaculture and livestock feed	[124,125]
Food/ Beverages	Natural colorants for sauce, dressing, emulsion oil, edible film, dairy products, bakery filling, confectionery, beverage, fortified juice, vegan sausage Prevention of lipid oxidation Lycopene: Natural red colorant, antioxidant ingredient in oil, edible film β-Carotene: Natural yellow–orange colorant in fat-based foods Lutein: Natural yellow to yellow-green colorant, functional foods	[110,125,126]
Nutraceutical/ Pharmaceutical	Lycopene: Ingredients for antioxidant, anticancer, cardiovascular health, and prostate health products β-Carotene: Provitamin A source in antioxidant support formulation, strengthening immunity, cardiovascular and eye health Lutein: Eye health ingredient; supporting macular pigment, protecting retina from blue-light and oxidative damage in eye	[18,127]
Cosmetics	Lycopene, β-Carotene, and Lutein: Photoprotection, anti-photoaging, antioxidant skin care ingredient Phytoene/phytofluene: Colorless carotenoids for UV protection, antioxidant support, skin health products	[128]

Future research should focus on linking carotenoid profiles with scalable recovery and stabilization processes. Specifically, establishing a feasible production platform requires an integrated strategy that combines genotype-based cultivar selection, metabolite profiling, green extraction technology, encapsulation systems, and techno-economic assessment (TEA). First of all, with respect to resource selection, multi-omics analysis combining genomics, transcriptomics, metabolomics, and carotenoid profiling can help to identify cultivars that can reliably produce carotenoids [2,9,11]. Simultaneously, synthetic biology and genome-editing approaches can redesign carotenoid metabolic fluxes for cultivar development [26,85,86,87,88]. In addition, the value of tomato pomace should be verified by validating its utilization routes and processing suitability to maximize its utilization for industrial raw material [18,28,29]. From an economic point of view, pomace is a low-cost raw material produced in large quantities during processing [17,18]. However, its economic feasibility depends on the seasonality of tomato harvesting, the cost of drying and storage, investment in advanced technologies, extraction yield, and product stability [23]. Future commercialization should therefore balance environmental impact, regulatory compliance, and market value by combining byproduct utilization with TEA-based process design [16,24].

## 7. Conclusions

Tomato cultivars exhibit substantial diversity in fruit color and carotenoid composition. Their characteristics are mainly governed by cultivar-specific genetic background, ripening stage, and metabolic regulation. Variations in key carotenoid biosynthetic genes, including *PSY1*, *CRTISO*, *LCY-B*, *LCY-E*, and *CYC-B*, result in distinct carotenoid profiles. Lycopene is predominant in red and pink cultivars, orange types are rich in β-carotene, and relatively low carotenoid accumulation is observed in certain yellow cultivars. Accordingly, fruit color can serve as a practical phenotypic indicator of carotenoid composition and underlying genetic variation. From an industrial perspective, the efficient utilization of tomato-derived carotenoids requires an integrated approach that links cultivar selection, biosynthetic regulation, byproduct valorization, process optimization, and product stabilization. In particular, the selection of raw materials based on cultivar-specific metabolic characteristics is essential for maximizing carotenoid recovery and quality. Conventional extraction methods remain useful, while emerging technologies, including ultrasound-assisted, microwave-assisted, enzyme-assisted, high-pressure-assisted, supercritical fluid, and deep eutectic solvent-based extraction methods can improve efficiency, sustainability, and scalability. In this context, the utilization of pomace can increase sustainability and support the development of a circular bioeconomy. Encapsulation and other formulation approaches can increase product value by improving the stability and bioavailability of carotenoids. Precise cultivar selection and process optimization will further improve application efficiency.

## Data Availability

No new data were created or analyzed in this study. Data sharing is not applicable to this article.

## Abbreviations

The following abbreviations are used in this manuscript:

ABA	Abscisic acid
CHY-B	β-carotene hydroxylase
CHY-E	ε-carotene hydroxylase
CRTISO	Carotenoid isomerase
CRTR-B1/2	β-carotene hydroxylase 1/2
CYC-B	Chromoplast-specific lycopene β-cyclase
CYP97A/C	Cytochrome P450 enzymes
DES	Deep eutectic solvents
DMAPP	Dimethylallyl diphosphate
GGPP	Geranylgeranyl diphosphate
GGPPS	GGPP synthase
GMOs	Genetically modified organisms
HPAE	High-pressure assisted extraction
LCY-B	Lycopene β-cyclase
LCY-E	Lycopene ε-cyclase
LHCII	Light-harvesting complex II
MAE	Microwave-assisted extraction
MEP	Methylerythritol-4-phosphate
NADES	Natural deep eutectic solvents
NXS	Neoxanthin synthase
PDS	Phytoene desaturase
PSY	Phytoene synthase
PEF	Pulsed electric field
SDS	Sodium dodecyl sulfate
SFE	Supercritical fluid extraction
TEA	Techno-economic assessment
UAE	Ultrasound-assisted extraction
ZDS	ζ-carotene desaturase
ZEP	Zeaxanthin epoxidase
ZISO	ζ-carotene isomerase
